# Macroscopically Ordered Piezo‐Potential in All‐Polymetric Solid Electrolytes Responding to Li Anode Volume Changes for Dendrites Suppression

**DOI:** 10.1002/advs.202509897

**Published:** 2025-10-13

**Authors:** Shuang‐Feng Li, Min Zuo, Jia‐Ming Wang, Li‐Qiang Peng, Bing Du, Yan‐Fei Huang, Zhong‐Ming Li

**Affiliations:** ^1^ Guangdong Provincial Key Laboratory of New Energy Materials Service Safety Shenzhen Key Laboratory of Polymer Science and Technology College of Materials Science and Engineering Shenzhen University Shenzhen 518060 P. R. China; ^2^ West China Hospital/West China School of Medicine Sichuan University Chengdu 610041 China; ^3^ State Key Laboratory of Advanced Polymer Materials Sichuan University Chengdu 610065 China

**Keywords:** all‐polymeric solid electrolytes, Li anode volume change, lithium dendrites, macroscopically ordered piezo‐potential

## Abstract

Solid‐state polymer electrolytes (SPEs) address the safety issue of lithium‐metal batteries but fail to resolve dendrite growth caused by anode volume fluctuations. Piezoelectric poly(vinylidene‐co‐trifluoroethylene) [P(VDF‐TrFE)] nanofiber interphases with aligned dipoles are developed that generate macroscopically directional electric fields during lithium expansion. Strategically orienting the piezoelectric field against Li^+^ migration redirects deposition from dendrite tips to planar regions through potential gradient steering. This approach enhances lithium salt dissociation while suppressing anions movement, achieving both high ionic conductivity (5.0 × 10^−4^ S cm^−1^) and Li^+^ transference number (0.40). Symmetric Li cells achieve 3000 h stability at 0.2 mA cm^−2^ and 25 °C, surpassing non‐piezoelectric SPEs by 750%. LiNi_0.8_Co_0.1_Mn_0.1_O_2_ (NCM811)//Li full cells retain 96% capacity after 400 cycles at 0.5 C. Crucially, reversing the orientation direction of the piezo‐electric field nullifies these benefits, proving that the direction of the piezoelectric field, not mere piezoelectricity, governs dendrite inhibition. This work introduces a novel strategy for inhibiting dendrite growth by leveraging the directionally engineered piezoelectric field of polymers.

## Graphical abstract

A P(VDF‐TrFE) piezoelectric interlayer is developed for solid‐state Li‐metal batteries. Strategically orienting the piezoelectric field against Li^+^ migration redirects deposition from dendrite tips to planar regions through potential gradient steering. This results in uniform Li plating and preventing the growth of lithium dendrites. The Li//Li cells achieve over 3000 h cycle life at 0.2 mA cm^−2^.

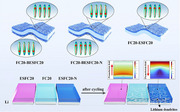

## 1 Introduction

Lithium metal's high theoretical capacity (3860 mAh g^−1^) and low electrochemical potential (‐3.04 V vs standard hydrogen electrode) position it as an ideal anode for next‐generation high‐energy‐density batteries.^[^ ^1^, ^2^, ^3^, ^4^ ^]^ However, the inherent phase‐conversion nature of lithium plating/stripping induces continuous dimensional fluctuations during cycling,^[^ ^5^ ^]^ generating cyclic stresses that mechanically fracture the protective solid electrolyte interphase (SEI).^[^ ^6^, ^7^, ^8^ ^]^ Such SEI damage persistently exposes fresh lithium surfaces to liquid electrolytes (LEs),^[^ ^9^ ^]^ accelerating LEs decomposition and side reactions at the anode‐electrolyte interface.^[^ ^10^, ^11^ ^]^ Ultimately, the growth of lithium dendrites would be induced,^[^ ^12^, ^13^ ^]^ which may penetrate separators, igniting flammable LEs and posing a risk of fire or even explosion to lithium metal batteries (LMBs).^[^ ^14^, ^15^ ^]^

To address the safety issue, one of the most promising methods is to replace LEs with chemically stable and nonflammable solid‐state electrolytes (SSEs).^[^ ^16^, ^17^, ^18^ ^]^ Solid‐state polymer electrolytes (SPEs),^[^ ^19^, ^20^, ^21^, ^22^ ^]^ particularly poly(vinylidene fluoride) (PVDF)‐based systems,^[^ ^23^ ^]^ show exceptional promise due to their intrinsic flexibility and processability alongside a wide electrochemical window. To date, most work focus on improving ionic conductivities of PVDF SPEs, such as dielectric modulation,^[^ ^24^, ^28^ ^]^ chemical modification,^[^ ^25^ ^]^ polymer blending,^[^ ^26^ ^]^ and 3D framework design.^[^ ^27^ ^]^ Notably, the dielectric modulation greatly enhanced the ionic conductivity to > 5.0 × 10^−4^ S cm^−1^.^[^ ^24^, ^28^ ^]^ However, despite these conductivity improvements, the current PVDF‐based SPEs are short of effectively suppressing dendrite growth, making LMBs suffer from poor cycling stability. Although some interfacial modification strategies have demonstrated partial success in dendrite suppression,^[^ ^29^ ^]^ they neglect the fundamental challenges of the lithium metal anode (LMA): its inherent cycling‐induced volume fluctuations persistently fracture the SEI and reignite interfacial degradation.^[^ ^5^, ^9^ ^]^ Therefore, it is of great importance, yet remains a great challenge, to develop a new strategy that not only improves ionic conductivities, but more importantly, responds to the huge volume changes of LMA to inhibit the growth of lithium dendrites.

In addition to serving as polymer matrices for SPEs, PVDF‐based polymers are also extensively studied as important piezoelectric materials.^[^ ^30^ ^]^ Owing to the piezoelectricity, PVDF‐based polymers show potential applications in the fields of actuators, sensors, medical monitoring, and energy harvesting.^[^ ^30^ ^]^ Since the large volume fluctuations of LMAs during cycling also exert stress on SPEs, causing them to generate piezoelectric potential, we speculate that piezoelectric PVDF‐based polymers should also have potential applications in the field of solid‐state LMBs. Nonetheless, such related study is rarely reported. Recently, research has explored using local piezo‐potentials to influence lithium‐ion migration or plating behavior in batteries.^[^ ^31^, ^32^ ^]^ However, these approaches primarily use piezoelectric ceramic fillers, which introduce problems like increased interfacial impedance, and uneven lithium‐ion distribution due to the filler agglomeration and conductivity mismatches with the matrix. This promotes non‐uniform lithium deposition and dendrite growth. More seriously, randomly oriented local piezo‐potential cannot bring interconnected pathways to directionally guide Li plating. These limitations prevent understanding how piezo‐potential direction governs Li deposition behavior and hinders effective dendrite suppression, resulting in poor battery cycling stability. Therefore, it is imperative to develop a new strategy capable of constructing a macroscopically ordered piezoelectric field without relying on inorganic ceramic fillers.

**Figure 1:**
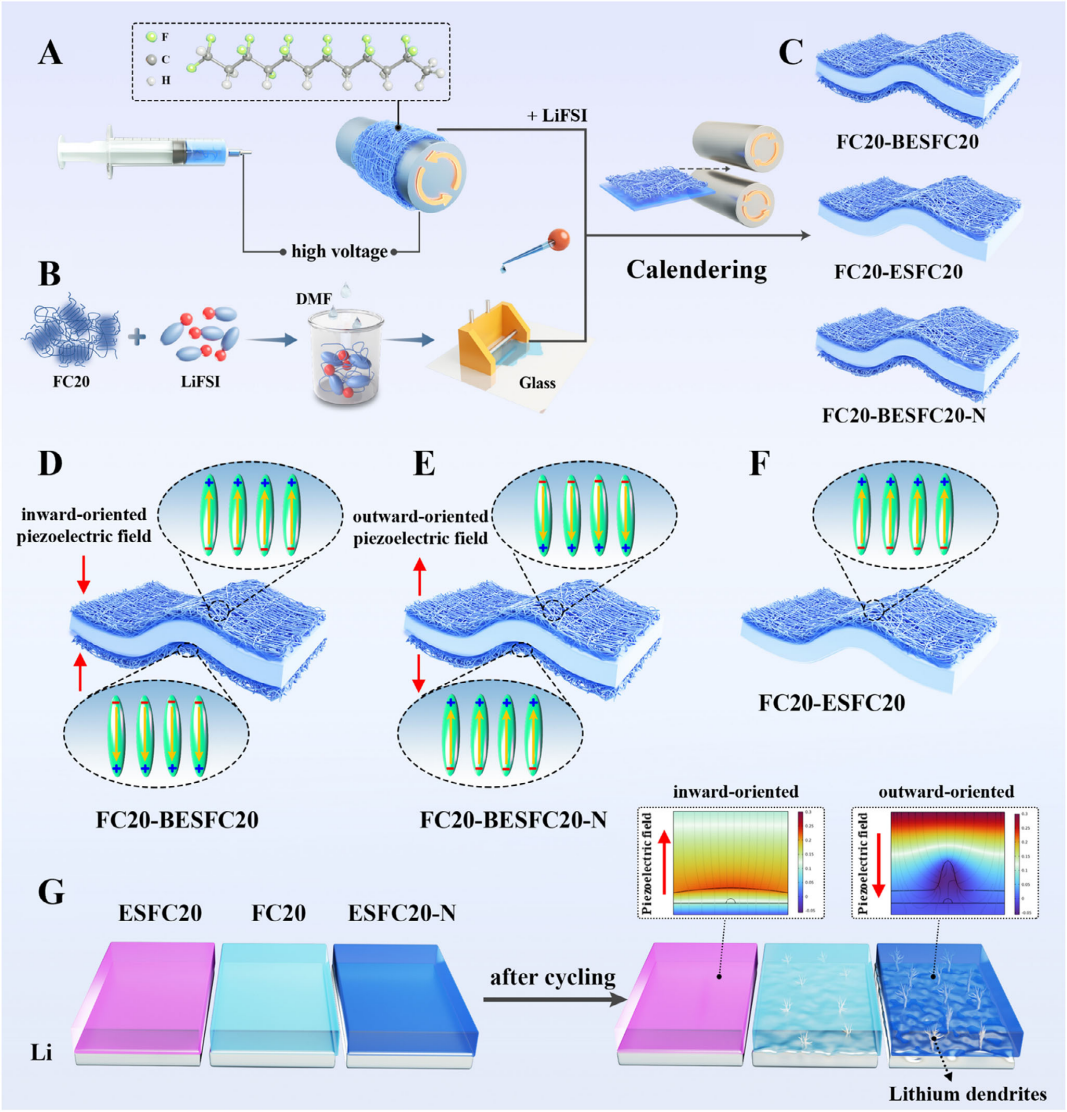
Schematic diagrams of the preparation methods. A) Using electrostatic spinning to obtain dipole oriented piezoelectric FC20 nanofiber film (ESFC20). B) Using the scratch coating method to prepare the normal FC20 electrolyte film. C) Piezoelectric ESFC20 electrolyte laminated with FC20 electrolyte by the calendering technique to form multilayered electrolytes. D–F) Schematic diagram of the dipole orientation direction of piezoelectric ESFC20 in different electrolytes. G) Schematic diagram of the mechanism by which piezoelectric effect inhibits lithium dendrite growth by adjusting the direction of lithium‐ion migration.

In this work, we construct a macroscopically ordered piezoelectric fields to respond to volume fluctuations of LMBs using a pure poly(vinylidene fluoride‐co‐trifluoroethylene), P(VDF‐TrFE), as the matrix of SPEs without adding any inorganic fillers (**Figure** **1**). Unlike randomly oriented local piezo‐potential,^[^ ^32^ ^]^ our macroscopic dipole alignment gives a high polarity to promote the dissociation of lithium salts while suppressing anion transport, increasing both the ionic conductivity and the lithium‐ion transference number (*t*_Li+_). More importantly, the inward‐oriented piezoelectric field (Figure 1D) redirects Li^+^ flux from dendrite tips to planar regions through potential gradient steering, thereby preventing the growth of lithium dendrites (Figure 1G). On the contrary, the outward‐oriented piezoelectric field (Figure 1E) accelerates the growth of lithium dendrites (Figure 1G), demonstrating the important role of the piezo‐potential direction, not merely piezo‐potential presence, governs dendrite inhibition. With a preferred direction of macroscopically ordered piezo‐potential, the Li//Li symmetric cells stably cycle for 3000 h and 1200 h at 0.2 and 0.3 mA cm^−^^2^, respectively, and LiNi_0.8_Co_0.1_Mn_0.1_O_2_ (NCM811)//Li cells exhibit 96% capacity retention after 400 cycles at 25 °C. In contrast, by reversing the direction of piezo‐potential, the Li//Li symmetric and NCM811//Li cells fail easily. This work pioneers the construction of macroscopically ordered piezo‐potential to transform volume changes of LMAs into dendrite‐suppressing stimuli, which hold great promise to prolong the cycling stability of LMBs.

## 2 Results and Discussion

**Figure 2:**
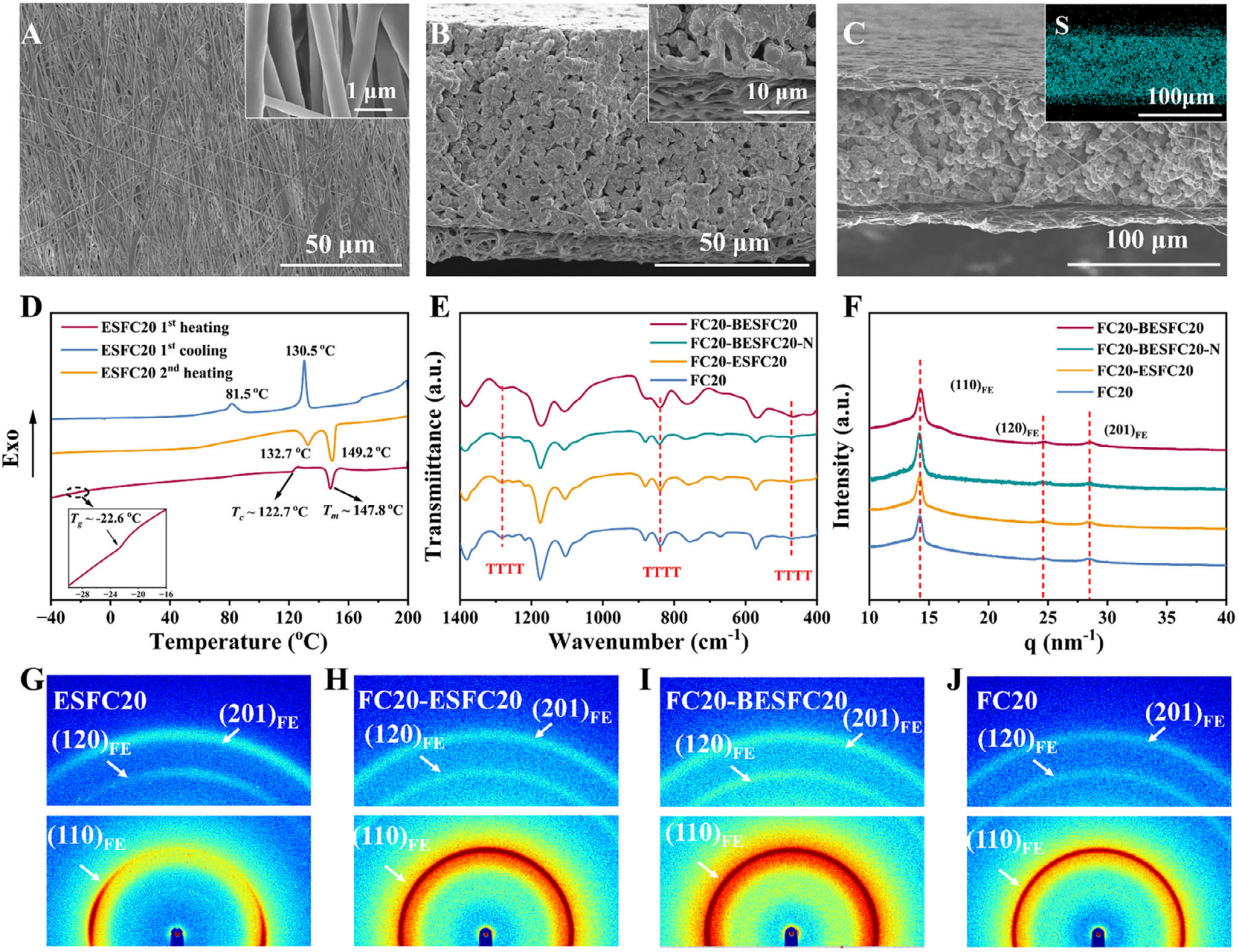
Structural characterization of ESFC20 and different electrolytes. A) SEM images of ESFC20. The cross‐sectional SEM images of B) FC20‐ESFC20 and C) FC20‐BESFC20, and the inset is energy dispersive spectroscopy (EDS) mappings of S. D) DSC curves of ESFC20. E) FTIR curves and F) XRD curves of different electrolytes. 2D WAXS images of G) the piezoelectric ESFC20 interphase layer, H) FC20‐ESFC20, I) FC20‐BESFC20, and J) FC20.

First, the piezoelectric interphase layer is prepared by electrostatic spinning (ES) of P(VDF‐TrFE) (FC20) (Figure 1A). FC20 is chosen for its superior all*‐trans* (TTTT) conformation formation compared to PVDF.^[^ ^33^, ^34^, ^35^ ^]^ Then, LiN(SO_2_F)_2_ (LiFSI) is introduced to make it conduct lithium ions (Figure S1, Supporting Information). Parallelly, FC20 SPE is prepared by solution scratch coating (Figure 1B). Before it is completely dried, the piezoelectric ESFC20 SPE is laminated to prepare multilayer SPEs with different laminating ways (Figure 1C–F). From scanning electron microscope (SEM) observation, the ESFC20 film exhibits interconnected nanofiber networks (**Figure** **2**A), which become tightly fused with FC20 SPE through calendaring (Figure S2, Supporting Information), forming seamless interfaces (Figure 2B,C) that are critical for continuous ion transport. Differential scanning calorimetry (DSC) results show that the glass transition temperature (*T*_g_) and the Curie transition temperature (*T*_c_) of the piezoelectric layer are ‐22.6 °C and 122.7 °C (Figure 1D), respectively, indicating that the chain segments are mobile, and the ferroelectric (FE) phase (corresponding to TTTT conformation) exists at room temperature.^[^ ^25^, ^36^, ^37^ ^]^ Structural characterization confirms pure TTTT conformations across all samples, evidenced by Fourier transform infrared spectroscopy (FTIR) peaks at 470, 840, and 1280 cm^−1^ (Figure 1E), and X‐ray diffraction (XRD) patterns matching FE‐phase crystallographic planes (14.3, 24.8, 28.6 nm^−1^) (Figure 1F).^[^ ^25^ ^]^ While 2D wide‐angle X‐ray scattering (2D WAXS) shows molecular chain orientation in standalone ESFC20 (Figure 2G) from electrospinning‐induced alignment,^[^ ^38^, ^39^ ^]^ multilayer composites (FC20‐ESFC20/BESFC20, B represents bilayer) exhibit isotropic patterns (Figure 2H,I) due to signal dominance by the thicker, unoriented FC20 matrix (Figure 2J). This interfacial architecture simultaneously assures rich FE‐phase and macroscopical orientation of dipoles in the interphase layer through controlled layer assembly.

**Figure 3:**
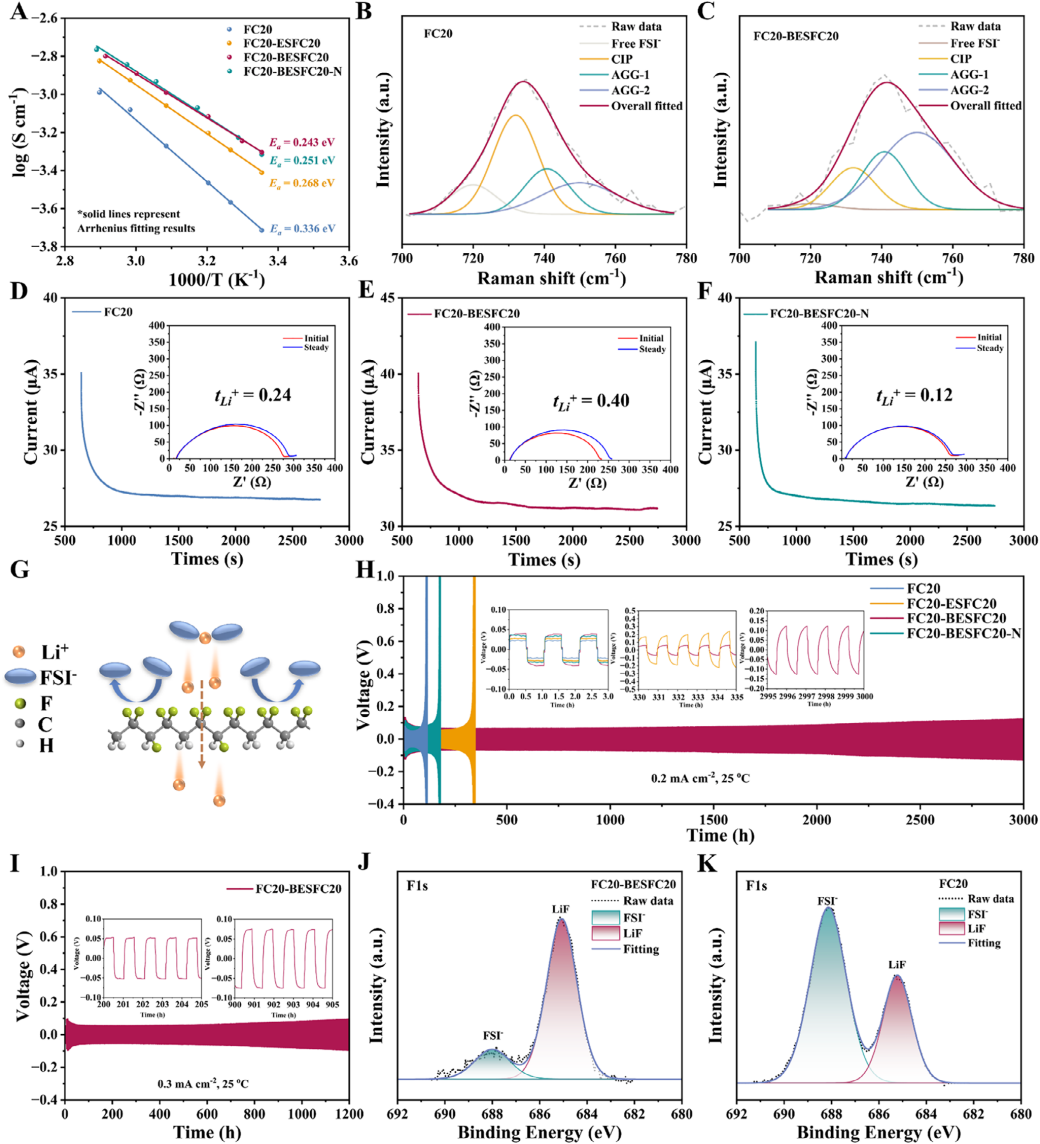
Characterization and analysis of electrochemical properties of different electrolytes. A) Ionic conductivities of various electrolytes at different temperatures and activation energy (*E*_a_) fitted according to the Arrhenius formula. Raman spectra of B) FC20 electrolyte and C) FC20‐BESFC20 electrolyte in the range of 700 to 800 cm^−1^. Li‐ion transference number of D) FC20 electrolyte, E) FC20‐BESFC20 electrolyte, and F) FC20‐BESFC20‐N electrolyte. G) Schematic diagram of the blocking effect of macroscopically oriented dipoles on the FSI^−^. H) Polarization voltage profiles of Li//Li symmetric batteries with various electrolytes at a current density of 0.2 mA cm^−2^ at 25 °C. I) Polarization voltage profiles of Li/FC20‐BESFC20/Li cell at a current density of 0.3 mA cm^−2^ at 25 °C. XPS spectra of the cycled lithium metal surfaces of Li//Li symmetric cells with different electrolytes after 50 h cycling under 0.2 mA cm^−2^: J) FC20‐BESFC20, K) FC20.

Electrochemical impedance spectroscopy (EIS, Figure S3, Supporting Information) was used to measure ionic conductivities. From **Figure** **3**A, adding a piezoelectric layer progressively enhances the conductivity from 1.9 × 10^−4^ S cm^−1^ (FC20 SPE) to 5.0 × 10^−4^ S cm^∓1^ (FC20‐BESFC20) at 25 °C, while reducing the activation energy to 0.243 eV. Given the comparable residual N, N‐dimethylformamide (DMF) content across different samples (Figure S4, Supporting Information) in the bound form (Figure S5, Supporting Information),^[^ ^40^, ^41^, ^42^ ^]^ the superior ionic conductivities of piezoelectric‐protected electrolytes are likely attributed to the oriented FE phase (containing macroscopically oriented dipoles, Figure 2G) that may promote the migration of ions.^[^ ^26^ ^]^ To prove this hypothesis, Raman test was performed. As shown in Figure 3B,C, the aggregated ion pairs (AGG) increase in piezoelectric‐protected FC20‐BESFC20 SPE (AGG‐1:24%, AGG‐2:55% vs 20% and 23% for FC20 SPE), which helps to form fast ion transport channels.^[^ ^43^, ^44^ ^]^ To further confirm this phenomenon, the *t*_Li+_ is calculated. From Figure 3D,E, the *t*_Li+_ rises from 0.24 (FC20 SPE) to 0.40 (FC20‐BESFC20 SPE), which proves again that the piezoelectric ESFC20 layer promotes the Li^+^ migration. Interestingly, the *t*_Li+_ decreases to 0.12 when dipole orientation reverses (FC20‐BESFC20‐N, N represents negative) (Figure 3F). This directional sensitivity stems from macroscopic piezoelectric field effects. That is, the oriented dipoles in FC20‐BESFC20 electrostatically impede bulky FSI^−^ anions while accelerating Li^+^ migration (schematically shown in Figure 3G).

The FC20‐BESFC20 electrolyte achieves the highest critical current density (CCD) in Li//Li symmetric cells (Figure S6, Supporting Information) and exhibits exceptional cycling stability of 3000 h at 0.2 mA cm^−2^ and 1200 h at 0.3 mA cm^−2^ at 25 °C (Figure 3H,I), which is 7.5 times longer than FC20 controls (Figure 3H). This stability stems from the piezoelectric interphase layer's ability to suppress Li dendrites, as confirmed by the cycled LMAs surface, where FC20 cells show dendritic protrusions (Figure S7A, Supporting Information) while FC20‐BESFC20 yields smooth, dendrite‐free surfaces (Figure S7B, Supporting Information). X‐ray photoelectron spectroscopy (XPS) reveals LiF‐dominated SEI layers in FC20‐BESFC20 (Figure 3J), which is known to enhance interphase stability,^[^ ^9^, ^45^ ^]^ whereas FC20 forms decomposition‐rich surfaces (FSI^−^ peak dominated, Figure 3K). Moreover, XPS C1s from FC20‐BESFC20 based cells show reduced signals of CO_3_^2−^ and C‐N species in comparison to FC20 based cells (Figure S8, Supporting Information), demonstrating that the piezoelectric layer helps to suppress undesirable side reactions.

Notably, the directional dependence of piezoelectric fields emerges as the decisive factor in dendrite suppression (Figure S9, Supporting Information). As shown in Figure 3H, FC20‐BESFC20 with an inward‐oriented piezoelectric field (Figure 1D) achieves exceptional cycling stability (>3000 h), whereas FC20‐BESFC20‐N featuring an outward‐oriented piezoelectric field (Figure 1E) fails prematurely (<250 h) despite comparable mechanical strength (Figure S10, Supporting Information) and SEI chemistry (Figure S8E, Supporting Information). This stark contrast highlights that macroscopic field orientation, not just piezoelectricity itself, governs dendrite inhibition efficacy. To decouple piezoelectric effects from other factors, we annealed (A) FC20‐BESFC20 sample to eliminate its piezoelectricity while retaining mechanical/chemical properties (Figures S10 and S8F, Supporting Information). The resulting FC20‐BESFC20‐A exhibited much poorer performance (<800 h, Figure S11, Supporting Information) compared to that of the piezoelectric FC20‐BESFC20, proving that the built‐in piezoelectric field, rather than mechanical or chemical attributes, dominates dendrite suppression. Such a positive role of the piezoelectric layer in suppressing dendrites growth is impressive when compared with other reported works (Figure S12, Supporting Information).^[^ ^29^, ^46^, ^47^, ^48^, ^49^, ^50^, ^51^, ^52^, ^53^, ^54^ ^]^ In conclusion, the hierarchy of factors governing dendrite suppression is: macroscopic polarization (piezoelectric effect) ≫ mechanical property/chemical stability.

**Figure 4:**
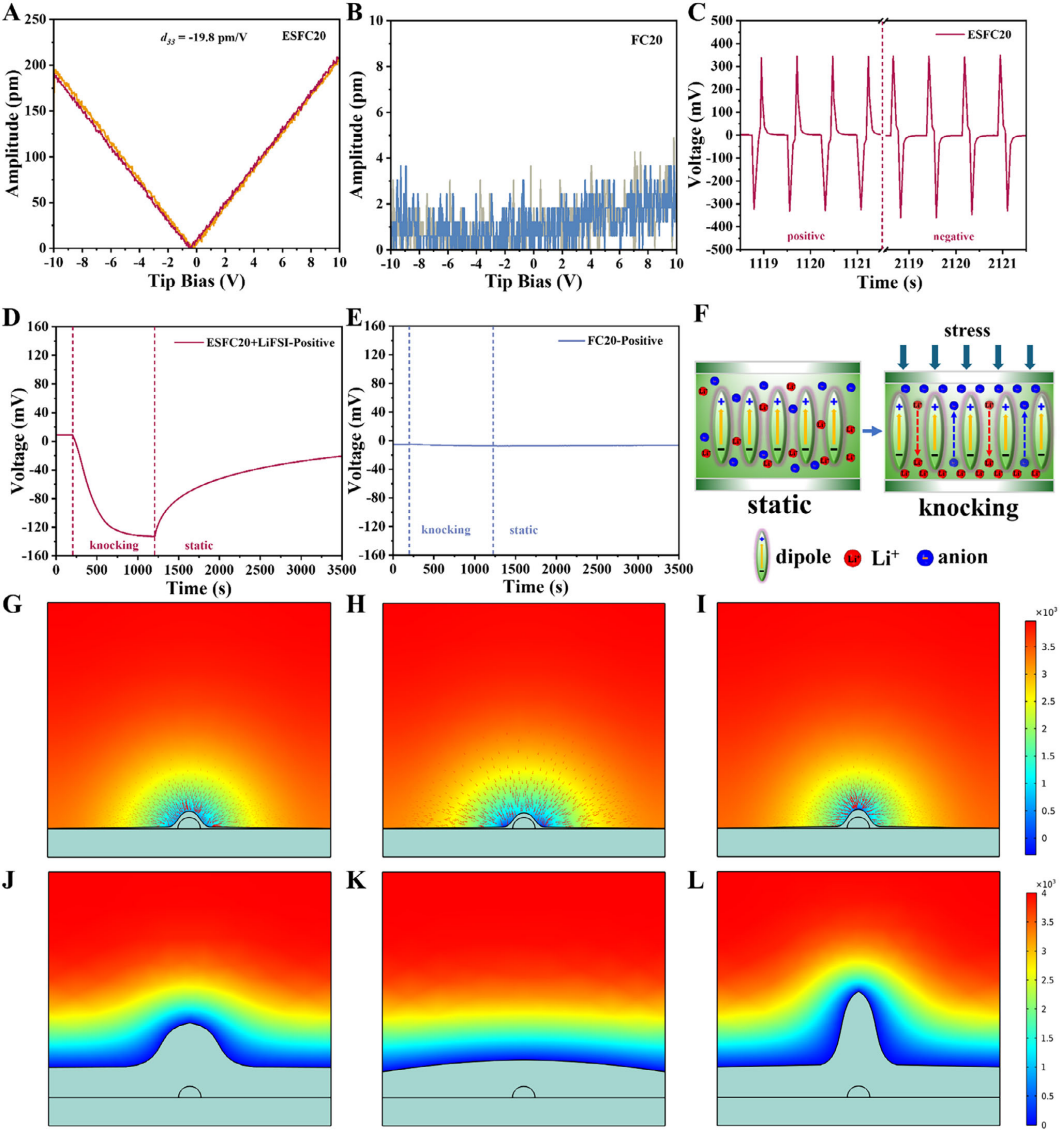
Characterization and simulation of piezoelectric behavior of the piezoelectric interphase layer. The amplitude–voltage curves of A) ESFC20 and B) FC20 in the PFM test. The voltage–time curves of C) ESFC20 in the dynamic piezoelectric test with open‐circuit voltage variation, containing both normal wiring and reversed positive–negative operating states. The curves of (D,E) positive wiring corresponding to open‐circuit voltage versus time when D) the stainless steel (SS)/ESFC20‐LiFSI/SS battery and E) the SS/FC20/SS battery are knocked by a force of 35 N. F) Schematic diagram of the ion distribution inside the battery during dynamic piezoelectric testing. Simulation of the dendrite growth by finite element field phase simulation in (G–I) initial and (J–L) final state: G,J) no piezoelectric field, H,K) the direction of the piezoelectric field same with lithium‐ion transport direction (marked by the red arrows), and I,L) the direction of the piezoelectric field opposite to the lithium‐ion transport direction.

To quantify the piezoelectric effect of the piezoelectric ESFC20 layer, the piezoelectric coefficient (*d_33_*) was determined by the piezoelectric response force microscopy (PFM) and dynamic piezoelectric test (see Figure S13, Supporting Information for test equipment diagram). PFM reveals *d*_33_ of ‐19.8 pm V^−1^ (calculated using Equation 4) for pure ESFC20 layer (contains no LiFSI, **Figure** **4**A), while unpolarized FC20 shows no response (Figure 4B). Dynamic testing of the ESFC20 film yields a *d*_33_ ≈ ‐8.6 pC N^−1^ (Figure 4C), calculated using Equation 5 based on the dielectric constant presented in Figure S14 (Supporting Information). The voltage response is comparable to that of commercial PVDF (Figure S15, Supporting Information), indicating good piezoelectric performance of ESFC20. In contrast, the FC20 film exhibits a negligible signal for the dynamic piezoelectric test (Figure S16A, Supporting Information). Two varied *d*_33_ values of the ESFC20 film is because PFM probes nanofiber‐level piezoelectricity, whereas dynamic testing averages over the porous film. These results confirm ESFC20 generates a built‐in piezoelectric field under stress. Thus, during Li dendrite growth, the piezoelectric layer should locally respond to anode deformation.

To probe the piezoelectric field's role, we measured open‐circuit voltage (OCV) changes in SS//SS cells under mechanical stress (simulating cycling conditions). The SS/ESFC20‐LiFSI/SS cell shows a reversible ≈140 mV OCV shift upon knocking/removing force (Figure 4D), while non‐piezoelectric FC20 exhibits almost no response (Figure 4E). Reversing electrodes inverted the signal (Figure S16B,C, Supporting Information), demonstrating that the voltage change in Figure 4D is caused by the piezoelectric field, rather than the triboelectricity.^[^ ^55^, ^56^ ^]^ The mechanism of such piezoelectrically driven voltage change might be explained as follows: i) Stress‐induced piezoelectric field drives ion migration, increasing OCV; ii) ion accumulation near electrodes counteracts the field (schematically shown in Figure 4F), stabilizing OCV; iii) force removal triggers rapid ion redistribution, dropping OCV. In summary, the ESFC20 with piezoelectric properties can effectively regulate ion transport behavior.

To investigate whether such changes in ion transport behavior affect the growth of lithium dendrites, multi‐physics field finite element simulations were carried out. As for the FC20 electrolyte (non‐piezoelectric), Li^+^ preferentially deposits at dendrite tips due to concentrated current lines (Figure S17A—C, Supporting Information), accelerating dendrite growth (Figure 4G; Figure S18A, Supporting Information; Figure 4J). As for the FC20‐BESFC20 electrolyte (piezoelectric), the piezoelectric field opposes the intrinsic electric field, flattening the potential difference. Current lines redistribute away from tips (Figure S17D—F, Supporting Information), guiding Li^+^ to flat regions and suppressing dendrites (Figure 4H; Figure S18B, Supporting Information; Figure 4K). As for the FC20‐BESFC20‐N electrolyte, the aligned piezoelectric field same as the original electric field enhances tip‐focused Li^+^ deposition (Figure S17G—I, Supporting Information), accelerating the dendrites growth (Figure 4I; Figure S18C, Supporting Information; Figure 4L). For short, the piezoelectric field direction critically determines dendrite inhibition efficacy. Opposing the intrinsic field (FC20‐BESFC20) is optimal for stabilizing Li metal anodes.

**Figure 5:**
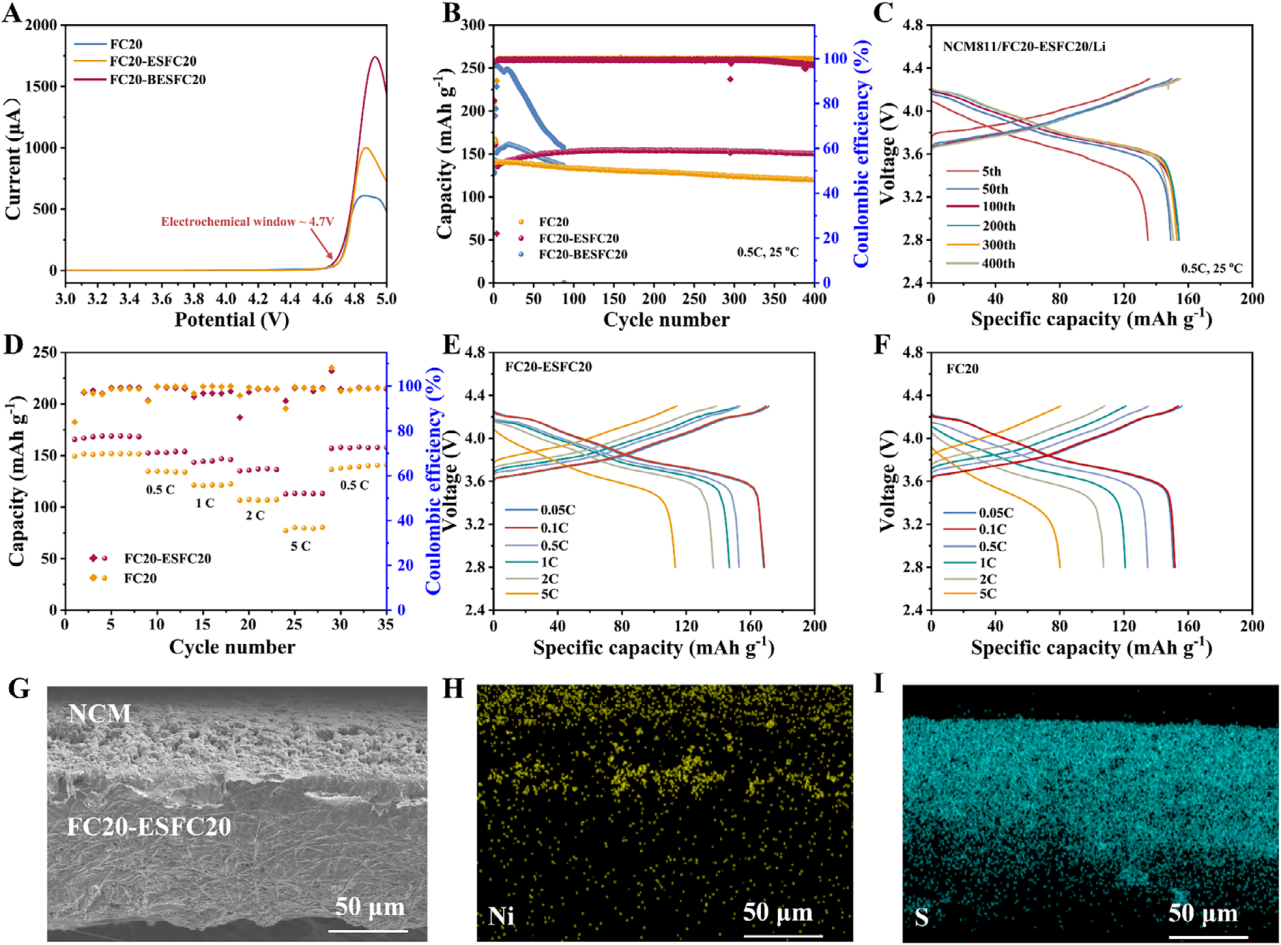
Characterization and analysis of electrochemical properties of piezoelectric interphase layer on full cells. A) Linear sweep voltammetry (LSV) curves of Li//SS batteries with various electrolytes. B) Cycling charge–discharge curves of various NCM811/Li cells at 25 °C and 0.5C. C) Voltage‐discharge specific capacity profiles of NCM811/FC20‐ESFC20/Li at 25 °C and 0.5C. D) Cycling charge–discharge curves of NCM811/FC20‐ESFC20/Li and NCM811/FC20‐ESFC20/Li at 25 °C and different rates. Voltage‐discharge specific capacity curves of E) NCM811/FC20‐ESFC20/Li and F) NCM811/FC20/Li at different multiplicities. G) SEM image of the interface between FC20‐ESFC20 and NCM811 positive electrode, along with the corresponding energy dispersive spectroscopy (EDS) mappings: H) Ni element distribution and I) S element distribution.

The piezoelectric protective layer's effectiveness was further evaluated in full cells using high‐voltage NCM811 cathodes. Linear sweep voltammetry (LSV) measurement shows that both FC20 SPE and its piezoelectric‐protected counterpart exhibit almost the same electrochemical stability windows of approximately 4.7 V (**Figure** **5**A), indicating the piezoelectric layer's compatibility with high‐voltage operation. When implemented specifically at the lithium anode interface, the piezoelectric modification demonstrates remarkable performance enhancements. The NCM811/FC20‐ESFC20/Li cell delivers a discharge capacity of 155 mAh g^−1^ after 100th cycle at 0.5C compared to 134 mAh g^−1^ for the unmodified FC20 system, while maintaining 96% capacity retention after 400 cycles versus just 84% for the control sample (Figure 5B,C; Figure S19, Supporting Information). The rate performance improvements are equally significant, with the piezoelectric‐protected cell sustaining 152 mAh g^−1^ at 0.5C and 112 mAh g^−1^ at 5C, outperforming the control cell's 135 and 79 mAh g^−1^, respectively (Figure 5D–F). However, this beneficial effect was strictly position‐dependent. When the piezoelectric layer was placed both at the cathode and the anode interface, cell performance deteriorated completely (Figure 5B; Figure S20, Supporting Information) due to excessive interfacial impedance (Figure S21, Supporting Information) caused by the layer's high modulus (Figure S10, Supporting Information). SEM characterization reveals both FC20‐ESFC20 and FC20 SPEs maintain excellent interfacial contact with NCM811 (Figure 5G—I; Figure S22, Supporting Information), which results in good compatibility between SPE and cathodes. This could be  proved by cyclic voltammetry (CV) measurement (Figure S23, Supporting Information). These comprehensive results clearly demonstrate that strategic placement of the piezoelectric layer at the lithium anode interface simultaneously addresses dendrite suppression and maintains full cell performance, offering a promising pathway for stable LMBs operation.

## 3 Conclusion

We develop a piezoelectric interphase layer between SPE and Li anode in LMBs that dynamically regulates lithium‐ion transport through its intrinsic piezoelectric effect. The layer's precisely aligned ferroelectric phase enhances lithium salt dissociation and directional ion transport, achieving a superior ionic conductivity (5.0 × 10^−4^ S cm^−1^) and Li^+^ transference number (0.40) at 25 °C. Crucially, we discover that orienting the piezoelectric field opposite to lithium‐ion transport direction optimally suppresses dendrites, enabling uniform lithium deposition. This design, combined with the layer's mechanical robustness and chemical stability, yields exceptional cycling performance. The Li//Li symmetric cells demonstrate rather long longevity (3000 h at 0.2 mA cm^−2^), while NCM811/Li full cells maintain 96% capacity retention after 400 cycles. Our work pioneers a materials design approach that simultaneously exploits PVDF‐based polymers’ dual functionality as both piezoelectric materials and solid electrolytes, opening new avenues for advanced battery development.

## 4 Experimental Section

### Materials

P(VDF‐TrFE) 80/20 mol% (FC20, *M_w_* = 450 000 g mol^−1^) was provided by Piezotech, Arkema. LiFSI (99.9%) and PVDF (Solef 5130, *M_w_* = 1000000 g mol^−1^) were provided by Guangdong Canrd New Energy Technology Co., Ltd., China.

### Preparation Methods

The ESFC20 membrane was prepared by electrospinning a solution of 0.85 g FC20 in DMF/acetone (3:2 v/v) at 16 kV, with a 1.2 mL h^−1^ feed rate, 12 cm spinning distance, and 30–40% relative humidity. The collected fibers were dried at 55 °C for 16 h to remove residual DMF. To prepare the ESFC20 electrolyte, the membrane was calendered and soaked in a LiFSI/DMF/ethanol solution (0.7:0.95:11.84 wt ratio), followed by solvent removal.

For the multilayer electrolyte, FC20 and LiFSI (1:1 wt ratio) were dissolved in DMF, cast into a film, and partially dried at 55 °C for 1 h. The half‐dry FC20 SPE was laminated with the piezoelectric ESFC20 electrolyte and pressed to ensure adhesion before final drying (55 °C, 2 h). Pure FC20 electrolyte was prepared similarly but fully dried after casting.

Batteries were assembled using NCM811 cathodes (8:1:1:1 NCM811/Super P/PVDF/LiFSI, ≈1 mg cm^−2^ loading on Al foil) paired with Li metal anodes in CR2032 coin cells. Symmetric Li//Li and Li//SS cells were also fabricated for electrochemical testing.

### Characterization of the Materials

The morphology was observed by SEM (Hitachi TM400 and Hitachi SU‐70, Japan) at an operating voltage of 5 kV. XRD measurement (Rigaku SmartLab, Japan) was executed using Cu‐Kα radiation with a wavelength, *λ*, of 1.5418 Å. The scattering vector *q* was calculated from the equation: *q* = (4π sin *θ*)/λ, where *θ* is the half‐scattering angle. FTIR spectra (Thermo Fisher Scientific, USA) were collected on a Nicolet 6700 FTIR spectrometer in an attenuated total reflection (ATR) mode. TGA was conducted on a TGA 55 analyzer (TA Instruments, USA) from room temperature to 800 °C with a 10 °C min^−1^ ramping rate under an N_2_ atmosphere. Raman spectra were collected on a RENISHAW in Via Raman microscope (UK).

### Electrochemical Characterization of Solid‐State Electrolytes

The ionic conductivity (σ) of the electrolyte was calculated using Equation (1),

(1) $$\sigma =\frac{L}{RS}$$

where *L*, *R*, *S* is the electrolyte thickness, the impedance of the SS//SS symmetric cells, and the area of the SS electrodes (the diameter is 15.6 mm). EIS profiles were obtained from 7 MHz to 1 Hz with a 10 mV AC oscillation voltage on a PMC2000A multichannel electrochemical station (PARSTAT, American) from 25 to 80 °C. The activation energy, *E_a_*, was calculated from the Arrhenius equation:

(2) $$\sigma =\sigma_{0}e^{-\frac{E_{a}}{RT}}$$

where *σ*_0_ is the pre‐exponential factor.

The 𝑡_𝐿𝑖+_ was determined using chronoamperometry combined with EIS. Lithium symmetric cells were first characterized by EIS (7 MHz to 1 Hz, 10 mV amplitude) to obtain initial impedance (*R*_0_). A 10 mV DC polarization was then applied while monitoring the current decay from initial (*I*_0_) to steady‐state (*I*_S_) values. Final impedance (*R*_S_) was measured after polarization removal. The 𝑡*_𝐿𝑖+_* was calculated using the equation:

(3) $$t_{Li^{+}}=\frac{I_{s}\left(\Delta V-I_{0}R_{0}\right)}{I_{0}\left(\Delta V-I_{s}R_{s}\right)}$$

Linear sweep voltammetry (LSV) was performed on Li/SPEs/SS cells at 1 mV s^−1^ (0–6 V vs Li/Li⁺). Symmetric Li/SPEs/Li cells were cycled at 0.2–0.3 mA cm^−2^ (0.5 h per half‐cycle), while NCM811/SPEs/Li full cells underwent initial activation at 0.05C (3 cycles) followed by 0.5C cycling, all conducted at 25 °C using a LAND test system.

### Characterization of Piezoelectricity

The voltages applied during the PFM testing are in the range of ‐10 to 10 V, and the equations used to calculate the piezoelectric coefficients *d*_33_ are as follows:

(4) $$d_{33}=\frac{A-A_{0}}{V-V_{0}}$$

where *A*, *V*, *A*_0_, and *V*_0_ denote the amplitude and voltage of the PFM amplitude‐voltage curves and the values of amplitude and voltage at the intersection point, respectively.

For the dynamic test, the external force is 35 N and the frequency is 1.4 Hz. The piezoelectric coefficient can be calculated as follows:

(5) $$d_{33}=\varepsilon_{r}\varepsilon_{0}A_{2}U/dF$$

where *ε*_r_ is the relative permittivity, *ε*_0_ is the vacuum permittivity, *A*_2_ is the force area, *U* is the voltage, *d* is the thickness of the sample, and *F* is the applied force.

### Multiphysics Field Simulation Modeling

All calculations are based on COMSOL simulation and calculation software. Please refer to the Supporting Information for details.

## Conflict of Interest

The authors declare no conflict of interest.

## Author Contributions

Y.F.H. conceived the idea and supervised the project. Y.F.H. and S.F.L. designed the experiments and wrote the paper. S.F.L., M.Z., J.M.W., and L.Q.P. conducted the measurement and analyses the data. B.D. and Z.M.L. helped review the manuscript and provided some testing equipment.

## Supporting information



Supporting Information

## Data Availability

The data that support the findings of this study are available from the corresponding author upon reasonable request.
