# Supplementary material for: Macroscopically Ordered Piezo‐Potential in All‐Polymetric Solid Electrolytes Responding to Li Anode Volume Changes for Dendrites Suppression
Source: Adv Sci (Weinh). 2025 Oct 13;13(2):e09897. doi: 10.1002/advs.202509897 (PMC12786380; doi:10.1002/advs.202509897)
Supplement: Supplementary file 1 — Supporting Information [file ADVS-13-e09897-s001.pdf]

Supporting Information

Macroscopically Ordered Piezo-Potential in All-Polymetric Solid Electrolytes Responding to Li Anode Volume Changes for Dendrites Suppression

Shuang-Feng Li, Min Zuo, Jia-Ming Wang, Li-Qiang Peng, Bing Du, Yan-Fei Huang, Zhong-Ming Li*

Multi-physics field simulation modeling

Mass transport

In the electrolyte, the transfer of ions is driven by migration because of electric field and diffusion because of concentration gradient which are governed by the Nernst−Planck equation:

$$N_i = -D_i\left(\nabla c_{0,i} - z_i^* F c_{0,i}^* \nabla\Phi/RT\right) \tag{1}$$

Where N_i is flux, D_i, z_i and $c_{0,i}$ is the diffusion coefficient, charge and concentration of species i, respectively. F is the Faraday's constant, R is the ideal gas constant, T is the Kelvin temperature and Φ is the electrolyte potential. Meanwhile, the ions in the electrolyte follows the equation of conservation of mass and charge:

$$\partial c_i/\partial t + \nabla \times N_i = 0 \tag{2}$$

$$\sum i z_i/c_i = 0 \tag{3}$$

where c_i is the concentration, z_i is the valence of each species in the electrolyte.

Charge transfer

Basically, Li^+ are transported from the bulk electrolyte to the anode surface then reduced to Li-atom, and at the interface of the electrolyte and the anode, the deposition process of Li^+ can be described as the simplified reaction:

$$Li^+ + e^- \leftrightarrow Li$$

The electrochemical behaviors of Li^+ at the electrode-electrolyte interface could be described by the famous Butler-Volmer equation:

$$i_{loc} = i_{ex}\left[e^{\frac{\alpha_a F\eta}{RT}} - e^{\frac{-\alpha_c F\eta}{RT}}\right] \quad (4)$$

where i_{loc} is the local current density, which could be used to quantify the local reaction rate. η is overpotential, α_a and α_c are the anodic and cathodic charge transfer coefficients, respectively, and i_{ex} is exchange current density which is defined as the current density flowing equally in each direction at the reversible potential and can be used to characterize the ease of a reaction to occur, moreover, it is closely related to the electron transfer kinetics and the concentration gradient near the surface:

$$i_{ex} = i_e \prod_{i,v_j>0}\left(\frac{c_{Li^+}}{c_b}\right)^{\frac{\alpha_a v_j}{\eta_j}} \prod_{i,v_j<0}\left(\frac{c_{Li^+}}{c_b}\right)^{\frac{-\alpha_c v_j}{\eta_j}} \quad (5)$$

Therefore, the i_{ex} is greatly influenced by the c_{Li^+}/c_b which is also refers to the concentration gradient. Where c_{Li^+} and c_b is the concentration of Li^+ near the anode and in the bulk electrolyte, respectively, i_e is the current density to represent the kinetics of electrons, v_j is the stoichiometric coefficients, n_j is the number of electrons transferred and the η is the overpotential.

$$\eta = \phi_s - \phi_e - U_{eq} \quad (6)$$

where ϕ_s and ϕ_e is the solid phase and electrolyte phase potential, respectively, U_{eq} is the equilibrium potential of the reaction.

Piezoelectric effect

We utilized the positive piezoelectric effect of the material, which refers to the change in polarization of the material when subjected to mechanical stress. The Hooke's Law and the electrical behavior of materials are coupled to illustrate the piezoelectricity, as given in the following equations:

$$S = s \times T \quad (7)$$

$$D = \varepsilon \times E \quad (8)$$

where S is strain, s is compliance, and T is applied stress; D is the electric charge density displacement, E is electric field strength, ε is permittivity; Their linear relations can be described as follows:

$$S = s^E \times T + dE \quad (9)$$

$$D = dT + s^T \times E \tag{10}$$

Due to the deposition of lithium, piezoelectric materials experience a tensile strain rate of $\dot{\varepsilon}$. The local tensile strain at any point on the surface of lithium metal is related to the normal deposition growth rate v_n, Therefore, the strain rate of piezoelectric materials can be written as:

$$\dot{\varepsilon} = k \times v_n \tag{11}$$

where k is the surface curvature.

By combining the above equations, the strain and piezoelectric values of the piezoelectric material can be obtained. By solving for the electric field resulting from the piezoelectric effect, followed by the subsequent correction of the electrolyte phase potential in the Nernst-Planck equation, the coupling for the entire simulation is accomplished. The equation can be expressed by:

$$\nabla^2 \varphi = -\frac{F}{\varepsilon_0 \varepsilon_r}(z_+ c_+ - z_- c_- + \rho) \tag{12}$$

where ε_0 is the vacuum permittivity, ε_r is relative permittivity. ρ denotes the bund charge density on surface of the molecular piezoelectric thin layer due to the spontaneous polarization.

General setup

To simplify the calculation, the size of the computation domain is set to 9×10 μm. The surfaces of negative electrodes are applied with 0V electric potential, and the positive electrode surfaces are applied with 0.2V electric potential. The initial Li^+ ion concentration in the electrolyte is set to 4000 mol m^{-3}. In the calculation of the initial electric field distribution, in order to reflect the influence of the initial disturbance, some small protrusions were set on the surface of the material to represent the influence of the initial lithium dendrite nucleation point.

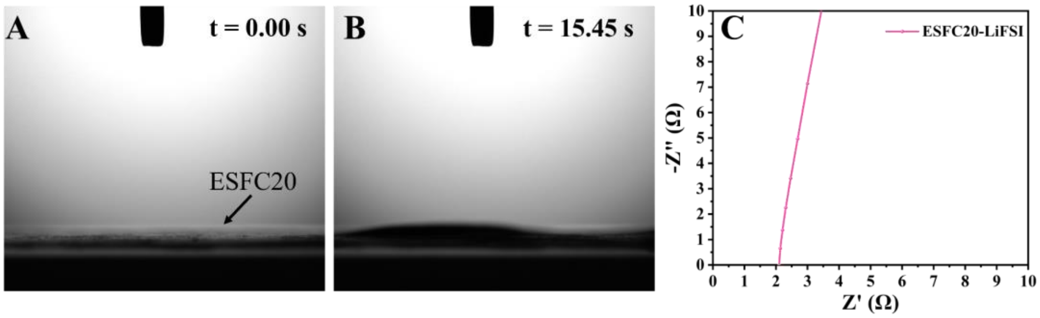

Figure S1. Preparation of the piezoelectric protective layer. The piezoelectric protective layer can be prepared by dropping a mixed solution of LiFSI with ethanol and DMF (ethanol : DMF = 15 ml : 1 ml) onto the electrostatically spun nanofiber membrane. Contact angle measurements (A) before and (B) after dropping the lithium salt solution, demonstrating that the lithium salt solution can infiltrate the ESFC20 well. (C) The piezoelectric protective layer (10 μm ~ 14 μm) can transport lithium ions with an ionic conductivity around 3.58 × 10^{-4} S cm^{-1}.

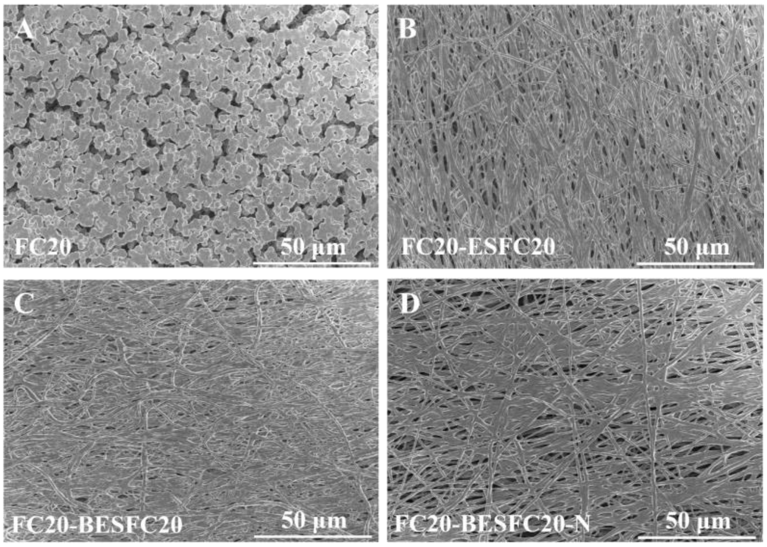

Figure S2. Surface SEM of different electrolytes. (A) FC20, (B) FC20-ESFC20, (C) FC20-BESFC20, and (D) FC20-BESFC20-N.

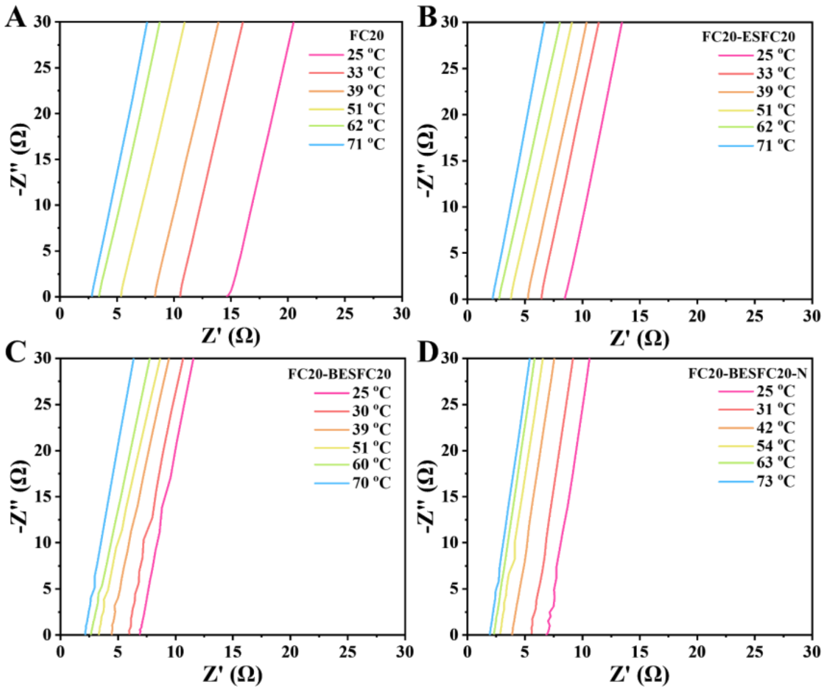

Figure S3. Nyquist plots of different electrolytes. EIS curves of (A) FC20 (thickness: L = 56 μm), (B) FC20-ESFC20 (L = 62 μm), (C) FC20-BESFC20 (L = 67 μm) and (D) FC20-BESFC20-N (L = 65 μm) sandwiched by two stainless plates of steel at varied temperatures.

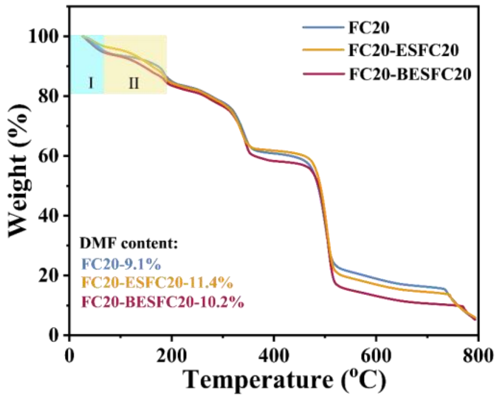

Figure S4. TGA curves of various electrolytes.

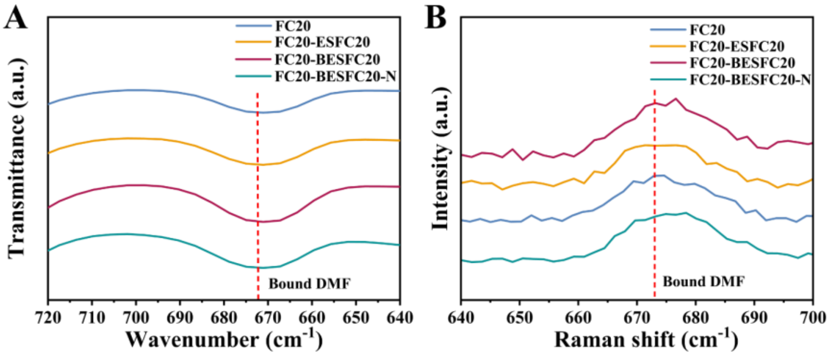

Figure S5. Characterization of bound DMF. (A) FTIR spectra of different electrolytes in the wave number range 720-640 cm^{-1}. (B) Raman spectra of different electrolytes in the wave number range 640-700 cm^{-1}.

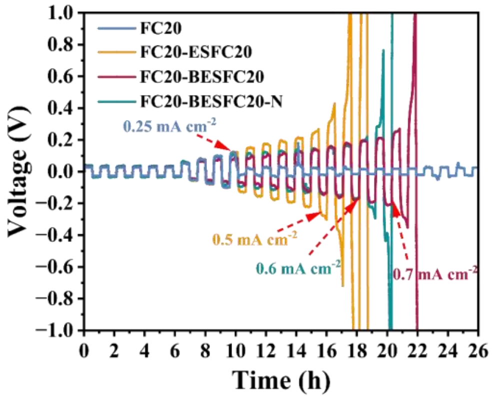

Figure S6. Comparison of critical current density of different electrolytes. Critical current density test with various electrolytes with a current difference of 0.05 mA cm^{-2} at each step.

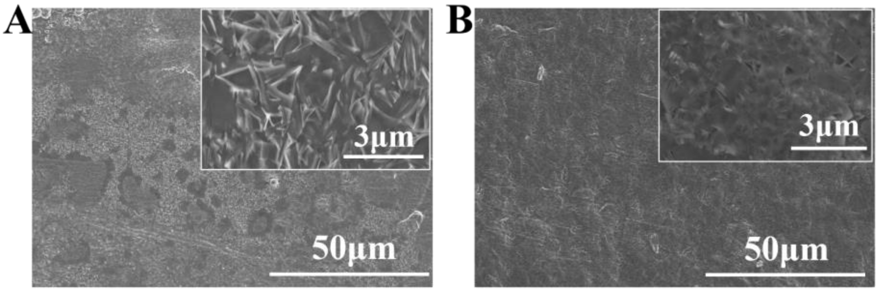

Figure S7. Surface morphology of cycled Li surface from (A) Li/FC20/Li and (B) Li/FC20-BESFC20/Li cells after 0.1 mA cm^{-2} cycling for 150 h.

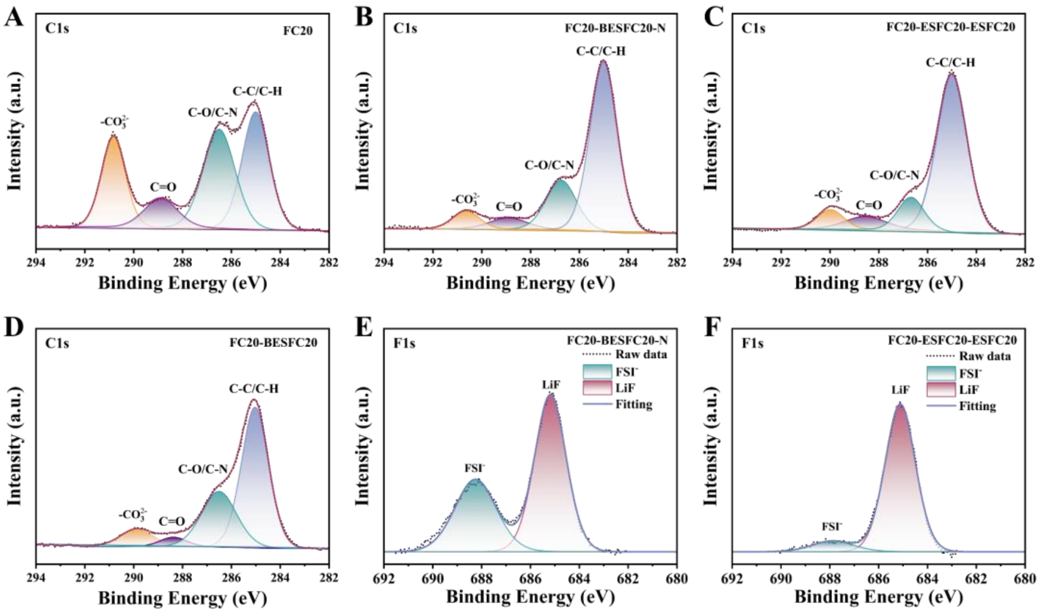

Figure S8. Characterization of SEI composition after cycling. XPS spectra of the corresponding lithium metal surfaces matching with different electrolytes after 50 h of cycling under 0.2 mA cm^{-2}. C1s spectra matching with (A) FC20, (B) FC20-BESFC20-N, and (C) the piezoelectric protection layer side of FC20-ESFC20, and (D) FC20-BESFC20. F1s spectra matching with (E) FC20-BESFC20-N, and (F) the piezoelectric protection layer side of FC20-ESFC20.

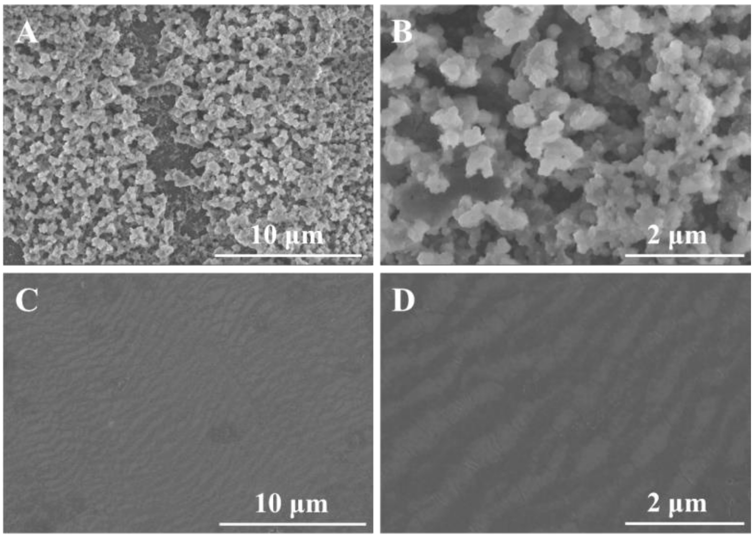

Figure S9. The inhibitory effect of different piezoelectric field directions on lithium dendrite growth. Morphologies of cycled lithium surface from (A and B) Li//FC20-

BESFC20-N//Li, and (C and D) Li//FC20-BESFC20//Li symmetrical cells after 0.2 mA cm^{-2} cycling for 100 h.

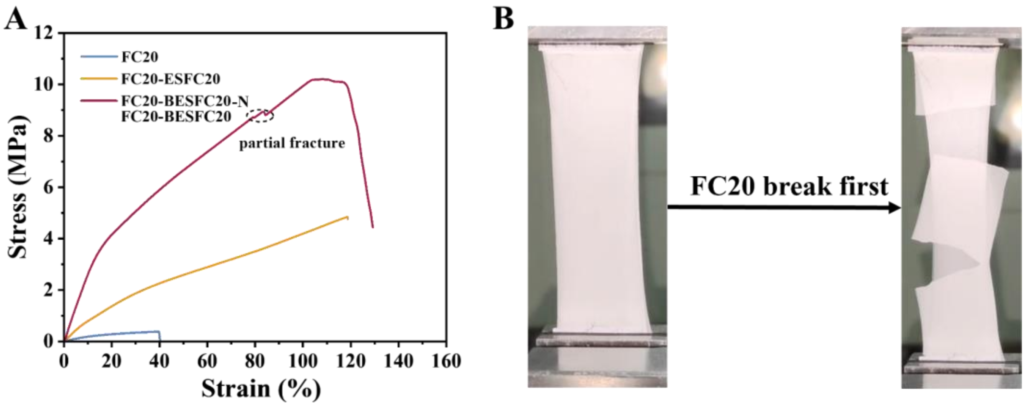

Figure S10. Characterization of mechanical properties of electrolytes. (A) Stress-strain curves of different electrolytes in tensile tests, (B) Digital photographs of FC20-BESFC20 in tensile with partial fracture of the middle layer.

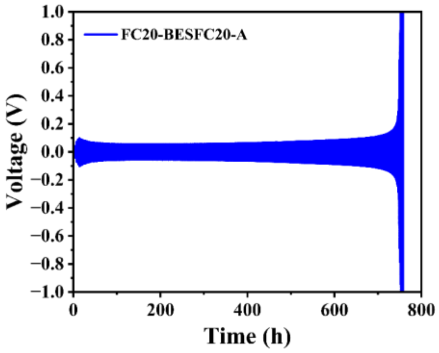

Figure S11. Polarization voltage profiles of Li/FC20-BESFC20-A/Li cells at a current density of 0.2 mA cm^{-2}.

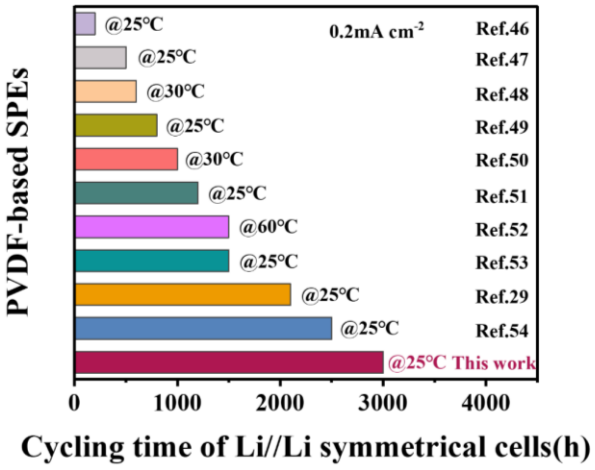

Figure S12. Cycling life of Li//Li symmetrical cells based on piezoelectric SPEs in this work and other PVDF-based SPEs reported in literatures.

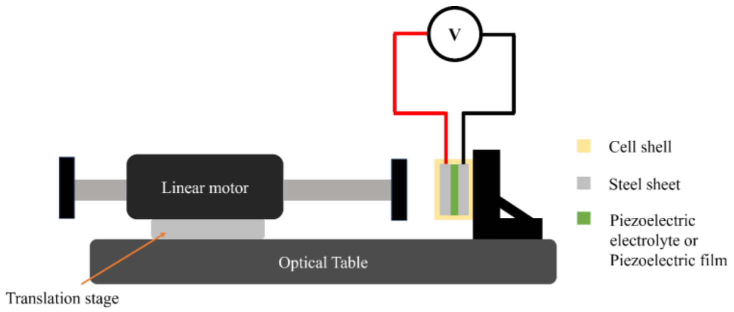

Figure S13. Schematic diagram of dynamic piezoelectric testing. The piezoelectric test employs a linear motor to apply force of specific frequency and magnitude (1.4 Hz, 35 N), and the generated voltage signals are recorded by an electrometer or electrochemical workstation.

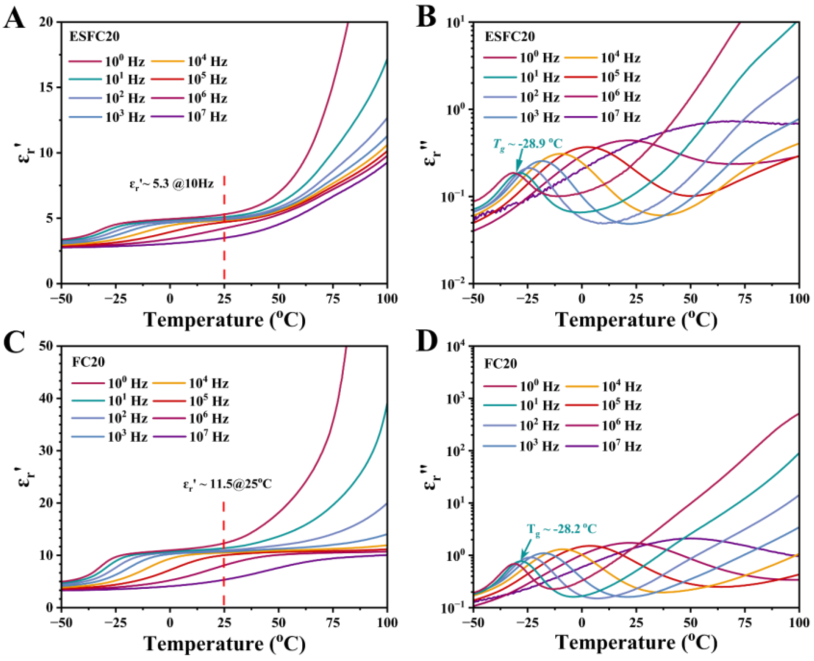

Figure S14. Dielectric measurements. Real part (ε_r') of relative permittivity as a function of temperature at different frequencies for (A) ESFC20 and (C) FC20 polymer films. The imaginary part (ε_r'') of relative permittivity as a function of temperature at different frequencies for (B) ESFC20 and (D) FC20 polymer films.

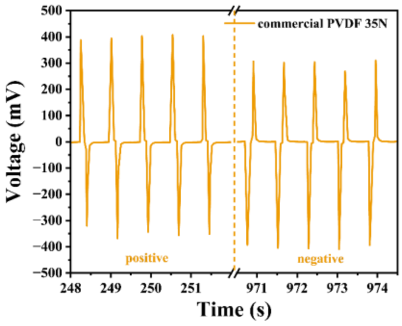

Figure S15. The dynamic piezoelectric testing of commercial piezoelectric PVDF.

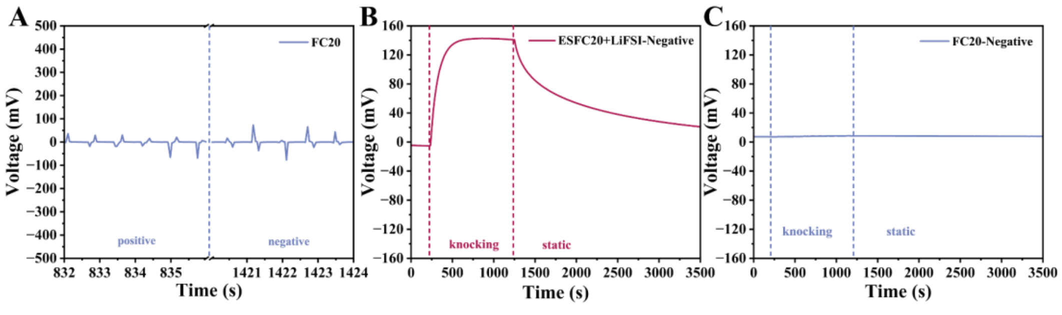

Figure S16. Dynamic piezoelectric testing. The voltage-time curves of (A) FC20 in dynamic piezoelectric test with open-circuit voltage variation, containing both normal wiring and reversed positive-negative operating states. The curves of (B and C) negative wiring corresponding to open-circuit voltage versus time when (B) the stainless steel (SS)/ESFC20-LiFSI/SS battery and (C) the SS/FC20/SS battery are knocked by a force of 35 N.

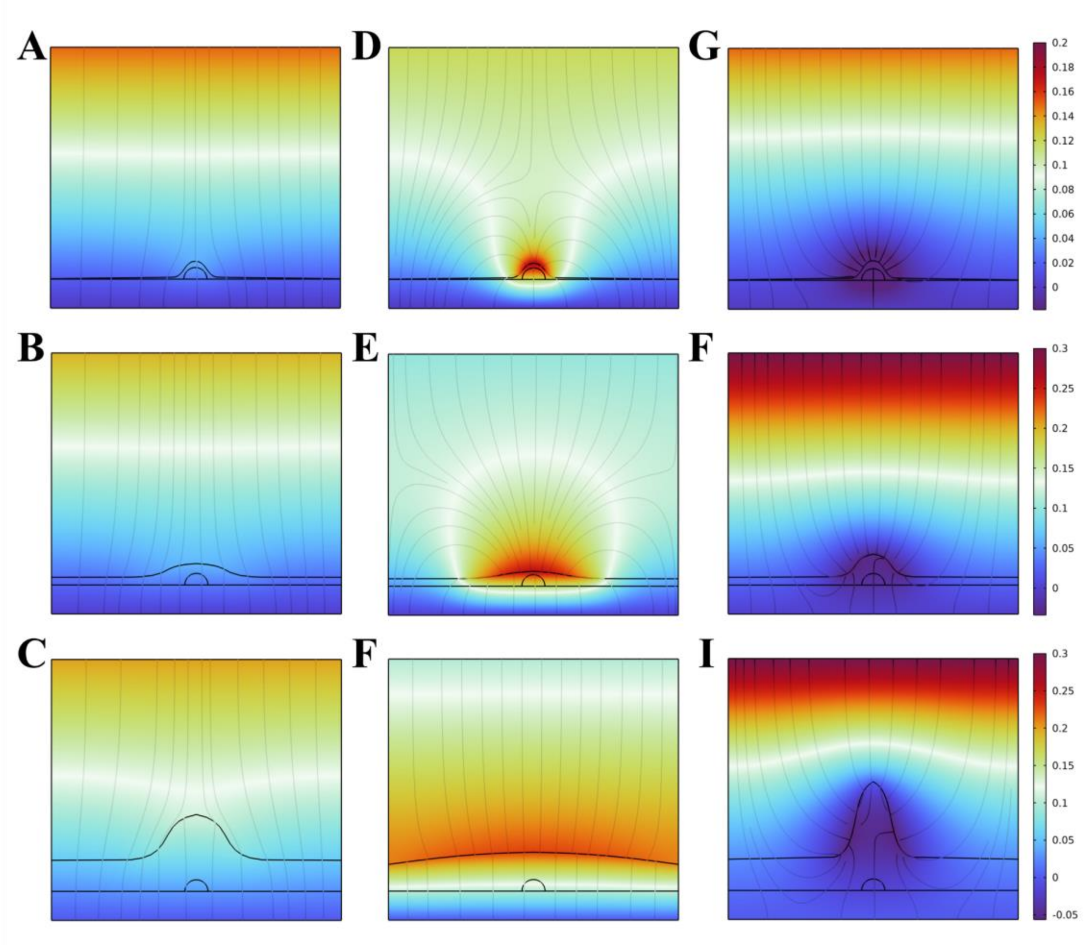

Figure S17. Presents finite element field simulations of the electric field changes around preset lithium protrusions under different piezoelectric field conditions and various lithium deposition times. The initial deposition state of lithium dendrite growth and the corresponding electric field conditions are shown, with black arrows indicating the current pathways. In (A to C), the piezoelectric field is set in the opposite direction to the initial electric field (lithium-ion transport direction), where A, B, and C correspond to the initial, intermediate, and final date, respectively. In (D to F), no

piezoelectric field is present, where D, E, and F correspond to the initial, intermediate, and final date, respectively. In (G to I), the piezoelectric field is set to be in the same direction as the initial electric field, where G, H, and I correspond to the initial, intermediate, and final date, respectively.

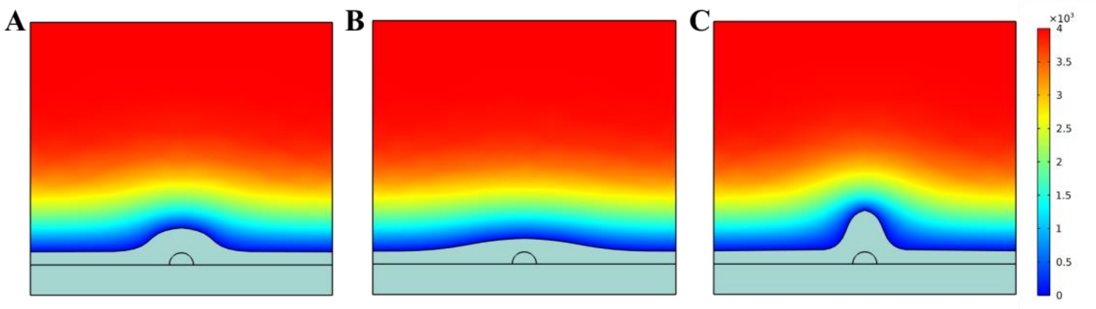

Figure S18. Simulation of the dendrite growth growth by finite element field phase simulation in intermediate date: (A) no piezoelectric field, (B) the direction of the piezoelectric field same with lithium-ion transport direction (marked by the red arrows), and (C) the direction of the piezoelectric field opposite to the lithium-ion transport direction.

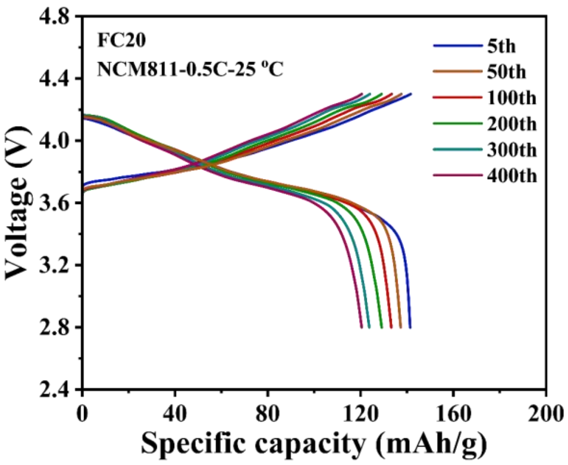

Figure S19. Voltage-discharge specific capacity curve of NCM811/FC20/Li at 25 °C, 0.5C.

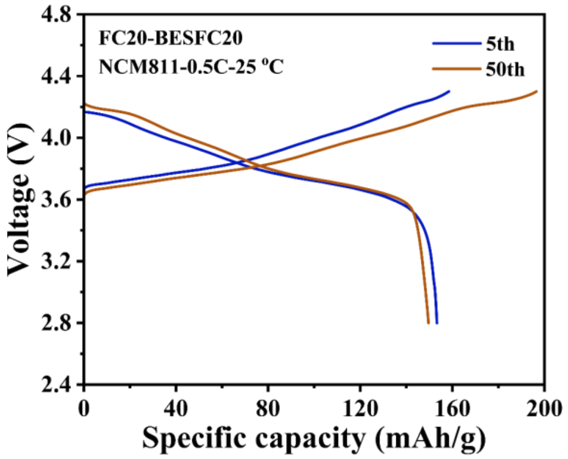

Figure S20. Voltage-discharge specific capacity curves of NCM811/FC20-BESFC20/Li at 25 °C, 0.5C.

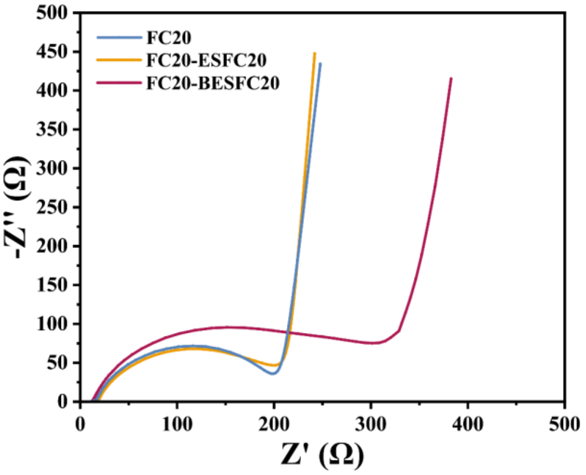

Figure S21. Electrochemical impedance spectroscopy (EIS) of the NCM811/FC20/Li, NCM811/FC20-ESFC20/Li, and NCM811/FC20-BESFC20/Li at 25 °C.

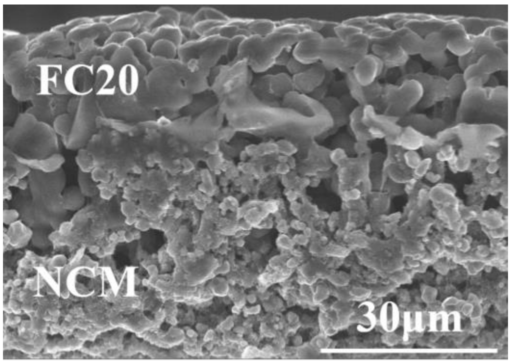

Figure S22. SEM image of the interface between FC20 and NCM811 cathode.

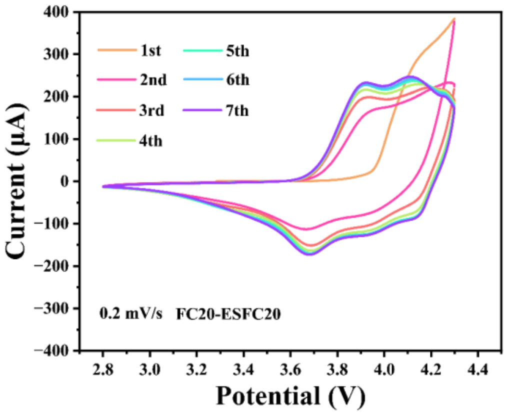

Figure S23. The cyclic voltammetry measurements of NCM811/FC20-ESFC20/Li cell (the voltage step is 0.2 mV/s).
